# Editorial: Cells, biomaterials, and biophysical stimuli for bone, cartilage, and muscle regeneration, volume II

**DOI:** 10.3389/fbioe.2024.1427256

**Published:** 2024-05-20

**Authors:** Lorenzo Fassina, Nora Bloise, Murugan Ramalingam, Livia Visai

**Affiliations:** ^1^ Department of Electrical, Computer and Biomedical Engineering, Centre for Health Technologies (CHT), University of Pavia, Pavia, Italy; ^2^ Department of Molecular Medicine, Centre for Health Technologies (CHT), INSTM UdR of Pavia, University of Pavia, Pavia, Italy; ^3^ UOR6 Nanotechnology Laboratory, Department of Prevention and Rehabilitation in Occupational Medicine and Specialty Medicine, ICS Maugeri IRCCS, Pavia, Italy; ^4^ Joint Research Laboratory (JRL) on Bioprinting and Advanced Pharma Development, A Joint Venture of TECNALIA and University of the Basque Country (UPV/EHU), Centro de Investigación Lascaray Ikergunea, Vitoria-Gasteiz, Spain; ^5^ NanoBioCel Group, Department of Pharmacy and Food Sciences, Faculty of Pharmacy, University of the Basque Country (UPV/EHU), Vitoria-Gasteiz, Spain; ^6^ IKERBASQUE, Basque Foundation for Science, Bilbao, Spain; ^7^ Bioaraba Health Research Institute, Vitoria-Gasteiz, Spain; ^8^ Biomedical Research Networking Centre in Bioengineering, Biomaterials and Nanomedicine (CIBER-BBN), Institute of Health Carlos III, Madrid, Spain

**Keywords:** tissue engineering, regeneration, bone, cartilage, muscle

Over the last few years, a variety of Tissue Engineering strategies have been developed to improve the regeneration of bone, cartilage, and skeletal muscle. Numerous studies have proven that physical factors (e.g., external forces, electromagnetic waves, electric fields, ultrasounds, lasers, fluid flow shear stresses, mechanical vibrations, mechanical deformations, and biomaterials’ features), as well as biochemical factors, may induce cells to reprogram their functions and dynamically adapt to the microenvironment conditions. In this context, many efforts are dedicated to engineer the biomaterial scaffolds, the physical stimuli, and the biochemical cues to whom the mammalian cells respond in terms of proliferation, differentiation, and production of extracellular matrix.

Effective regeneration of bone, cartilage, and skeletal muscle defects often presents significant challenges, particularly in patients with decreased tissue regeneration ability due to extensive trauma, diseases, or aging. To this regard, in the present Research Topic, 90 Authors from all over the world decided to publish their outstanding and promising results.

In particular, Wu et al. showed that, during the mineralization period of distraction osteogenesis in a rat model, the injection of rat bone marrow mesenchymal stem cells, which were differentiated by recombinant rat platelet-derived growth factor BB (rrPDGF-BB), can successfully promote the bone regeneration inside the distraction space.

Ren et al. made a comprehensive review regarding the action performed by the mesenchymal stem cells through their exosomes (MSC-Exos), thus highlighting that MSC-Exos are effective in promoting the osteogenesis.

In addition, Liu et al. thoroughly reviewed a growing evidence indicating that the microRNAs delivered by small extracellular vesicles originating from mesenchymal stem cells can enhance the bone regeneration.

Jaber et al. developed a powerful *in silico* approach to evaluate, in pre-clinical studies, the design of scaffolds for the bone regeneration within long bone large defects; their approach could lead to optimized architectures of 3D printed implants for bone regeneration. In particular, they simulated *in silico* that PCL strut-like scaffolds appear superior to gyroid ones in terms of bone regeneration despite their large surface curvatures.

Moreover, Sanaei et al. showed that, in endoprosthetic reconstruction surgery, reducing the prosthesis modulus by inclusion of an open-space lattice has a positive effect on bone tissue particularly within the periprosthetic zones; the improved mechanics appears to also have a positive effect on the osteointegration.

Gao et al. systematically reviewed the physiological mechanisms underlying the mandibular flexure, discussing different concurrent deformation types, moreover, they explored the deep implications of mandibular flexure on clinical aspects such as bone absorption around dental implants.

Dabaghi et al. evaluated the regeneration capability of a human-derived demineralized scaffold for the meniscal regeneration; overall, the results suggest that the new scaffold could be used as a promising biocompatible graft material for the meniscal tissue regeneration.

Luo et al. synthesized biocompatible RGD conjugated-sonosensitizer-nanoparticles to regulate the chondrogenic differentiation of bone marrow mesenchymal stem cells; such nanoparticles have the ability to generate a moderate level of ROS via an ultrasound treatment: this leads to an enhanced chondrogenic differentiation and to the buildup of cartilage extracellular matrix.

In addition, Qiang et al. showed that the sequential release of Bevacizumab (an inhibitor of the vascular endothelial growth factor) followed by insulin-like growth factor-1 (a cartilage repair factor), both delivered from microspheres contained in a hydrogel, can effectively improve the cartilage regeneration in a rabbit model of proximal tibial growth plate injury. In particular, they proposed a novel approach, where the inhibition of osteogenic differentiation and bone bridge formation is prioritized before promoting the chondrogenic differentiation.

On the other hand, Gao et al. studied, *in vitro*, the creep deformation of articular cartilage under the physiological loads occurring in daily activities such as standing, single-leg lunge, and the stance phase of gait; their *in vitro* model together with a viscoelastic constitutive law was employed to predict the creep-recovery behavior of the cartilage. If not fully recovered in time, the creep deformation may induce some damage in the cartilage; as a consequence, these findings could provide new understandings of normal joint function and cartilage pathology.

Mahdavi-Jouibari et al. focused on stem cells from human exfoliated deciduous teeth, which have a clear chondrogenic differentiation potential together with minimal immunogenicity and can be an interesting option for cartilage regeneration.

Finally, Wang et al. reviewed the role of macrophage polarization in tendon healing, focusing on insights from animal models; in particular, the review explores the complex role of macrophages in tendon pathology, detailing how various macrophage phenotypes contribute to both healing and adhesions’ formation. The review also searches the potential of modulating the macrophage activity to enhance the tendon repair and to minimize the adhesions.

